# Dietary DGLA-induced ferroptosis is suppressed by vitamin B12-mediated phosphatidylcholine synthesis

**DOI:** 10.70401/fos.2026.0045

**Published:** 2026-09-18

**Authors:** Madison S. Mortensen, Paige E. Silvia, Carissa Perez Olsen, Jennifer L. Watts

**Affiliations:** 1 School of Molecular Biosciences, Washington State University, Pullman, WA 99164, USA.; 2 Department of Chemistry and Biochemistry, Worcester Polytechnic Institute, Worcester, MA 01609, USA.

**Keywords:** Dihomo-gamma-linolenic acid, DGLA, lipids, ferroptosis, vitamin B12, phosphatidylcholine, germ cells, *C. elegans*

## Abstract

**Aims:**

Ferroptosis is a form of cell death associated with uncontrolled lipid peroxidation of polyunsaturated fatty acids (PUFAs) leading to membrane destruction. We previously showed that dietary dihomo-gamma-linolenic acid (DGLA) induces ferroptosis in *Caenorhabditis elegans* germ cells in a dose-dependent manner, and that the sensitivity of germ cells to dietary DGLA is altered by many genetic, dietary, and chemical interventions.

**Methods:**

The severity of germ cell death was documented after dietary supplementation of DGLA, vitamin B12, methionine, and choline to wild type and mutant *Caenorhabditis elegans* (*C. elegans*). The presence of germ cells, gametes, and embryos was visualized in young adult worms with 4′,6-diamidino-2-phenylindole (DAPI).

**Results:**

The addition of vitamin B12 rescued DGLA-induced cell death. For this protection, vitamin B12 functions as a cofactor for methionine synthase (METR-1). In addition, dietary methionine and choline rescued germ cell death in the context of low-B12 diets as well as in ferroptosis-sensitive *metr-1* and *sams-1* mutant strains. The *pcyt-1* mutant worms, with reduced capacity for phosphatidylcholine (PC) synthesis, were not responsive to dietary vitamin B12-mediated rescue of DGLA-induced ferroptosis, providing evidence that PC synthesis and homeostasis play key roles in ferroptosis susceptibility. Oleic acid supplementation rescues DGLA-induced cell death in wild type, but not in *pcyt-1* mutant strains.

**Conclusion:**

These studies reveal the requirement for dietary vitamin B12 and sufficient PC synthesis in protection from dietary DGLA-induced ferroptosis, highlighting the importance of phospholipid metabolism in ferroptosis regulation.

## 1. Introduction

Ferroptosis is an iron-dependent form of cell death whose main mechanism of action revolves around uncontrolled lipid peroxidation within the cellular membrane, causing damage and eventual cell death^[1,2]^. Ferroptosis has been implicated in a variety of human disorders and diseases, including neurodegeneration and iron overload syndrome^[3,4]^. Furthermore, ferroptosis has great potential in cancer and tumor treatment^[5–8]^. Important mechanistic details of ferroptosis have been revealed using cell culture systems; however, whole animal models are vital for studies examining dietary, environmental, and homeostatic aspects of ferroptosis.

*Caenorhabditis elegans* (*C. elegans*) is a nonparasitic nematode that, when grown experimentally, feeds on a bacterial lawn typically comprised of a nonpathogenic strain of *E. coli*^[9]^. The worm and its bacterial food source comprise a two-species system, where the dietary bacteria directly impact the physiology of the worm. As such, *C. elegans* allows for accessible and powerful studies on the effects of specific nutrients obtained from the diet^[10–12]^. The difference between worms grown on various bacteria has been extensively documented, including changes in fat content^[10,13]^, the microbiome^[14–16]^, and fertility^[17]^. Notably, previous literature demonstrated that cobalamin, or vitamin B12, varies significantly depending on the strain of dietary bacteria fed to the worms^[12,18–21]^.

We previously documented that the dietary supplementation of the polyunsaturated fatty acid (PUFA) dihomo-gamma-linolenic acid (DGLA) induces germ cell death in *C. elegans* compared to equivalent doses of other supplemented fatty acids^[22]^. The DGLA supplementation increased the DGLA levels in all major lipid classes in the worm^[22]^. The cell death was prevented by the radical trapping small molecule inhibitor ferrostatin-1 and germ cell death was enhanced in *C. elegans* mutant strains with reduced glutathione peroxidase and ferritin activities^[23]^. Additionally, DGLA was also found to be sufficient to induce ferroptosis in HT-1080 human cancer cells^[23]^. Therefore, death of germ cells in young *C. elegans* treated with DGLA represents a physiological, whole animal model of ferroptosis triggered by dietary modification in the absence of drugs that interfere with the function of glutathione antioxidant systems or iron overload.

Our study explored how critical metabolites affect the susceptibility of germ cells to DGLA-induced ferroptosis. To determine the reasons for the difference in dietary DGLA responses in wild type worms fed on two different *E. coli* diets, we found that dietary vitamin B12 conferred resistance to dietary DGLA-induced ferroptosis in *C. elegans*. Furthermore, we identified methionine synthase, which utilizes vitamin B12 as a cofactor, as the key enzyme that mediates this resistance. We also identified dietary methionine, the product of methionine synthase, and dietary choline, a component of phosphatidylcholine (PC), as strong suppressors of DGLA-induced ferroptosis. We found that vitamin B12 was unable to suppress ferroptosis in a mutant strain with impaired PC synthesis. These findings highlight that the synthesis and maintenance of PC is an important aspect of ferroptosis modulation.

## 2. Methods

### 2.1 *C. elegans* strains and maintenance

All *C. elegans* strains were maintained on standard nematode growth medium (NGM) agar plates seeded with a pre-cultured common laboratory bacterial strain *E. coli* OP50 at 20 °C unless otherwise stated. The following *C. elegans* strains were used in this study: wild type (WT) N2 Bristol; RB755 *metr-1(ok521)* and RB1434 *mmcm-1(ok1637)*, both of which were provided by the Caenorhabditis Genetics Center, which is funded by the National Institutes of Health (NIH) Office of Research Infrastructure Programs (P40 OD010440). The HA1947 *sams-1(ok3033)* strain was a gift from Dr. Amy Walker at the University of Massachusetts Chan Medical School, and the *pcyt-1(et9)* (C211Y) strain was a gift from Dr. Marc Pilon at the University of Gothenburg. The *metr-1(ok521)* strain was outcrossed 4x against N2.

### 2.2 Supplementation of dietary nutrients

NGM media was supplemented with Tergitol (Sigma-Aldrich Cat#NP40S) at a final concentration of 0.1% and various doses of DGLA sodium salt (NuChek, Cat #S-1143) dissolved in water before seeding with OP50^[24]^. Similarly, Vitamin B12 (cyanocobalamin, Sigma-Aldrich, Cat #V2876), L-Methionine (Sigma-Aldrich, Cat #M9625), and choline chloride (Thermoscientific Cat#110290500) were diluted in water and added to NGM media containing 0.1% Tergitol.

### 2.3 *C. elegans* sterility analysis

Embryos from populations of wild type or mutant *C. elegans* were isolated by the hypochlorite method and allowed to hatch overnight in M9 buffer. The synchronized L1 larvae were plated onto treatment or control plates and incubated 3–4 days at 20 °C. Upon reaching adulthood, a population of 200 wild type or mutant *C. elegans* was observed under a dissecting microscope. Because *C. elegans* are transparent, it is possible to observe the presence or absence of embryos within the uterus of each worm once they reach egg-producing age, indicative of fertility or sterility, respectively. For technical replicates, a total of 50 young adult worms were observed and their fertility state recorded. Statistical analysis is shown in Data S1.

### 2.4 *C. elegans* germline morphology analysis

Whole mount fixation of young adult nematodes that were treated and grown as described in Section 2.3 was performed using Carnoy’s fixation (six parts acetic acid, 3 parts 100% ethanol, 1 part chloroform). Worms collected in 5 μL of M9 buffer were applied to a glass slide and 10 μL Carnoy’s fixative was added. After fixation, the slides were air dried and stored at 4 °C until stained. The fixed worms were rehydrated with 20 μL M9 for 30 minutes. Immediately afterwards, the worms were resuspended in a 1:1 mixture of Vectashield (Vector Laboratories Cat #H-1000–10) and 4′,6-diamidino-2-phenylindole (DAPI) solution (1.7 μg of DAPI in M9 buffer (Cayman Chemical Cat #28718–90-3)). After allowing the DAPI to penetrate for at least 30 minutes obscured from light, slides were stored at 4 °C.

*C. elegans* stained with DAPI were imaged on an Olympus BX53 fluorescent microscope. Individual worms from each treatment group were classified into one of four possible groups based on germline morphology. Normal (fertile) worms had two gonadal arms, gametes (sperm and oocytes), and visible embryos. Abnormal (fertile) worms had one or two deformed or absent gonadal arms, some gametes and embryos present in the uterus. Abnormal (sterile) worms displayed severe deformity or absence in one or both gonadal arms and did not produce embryos. Absent (sterile) worms were classified by a complete absence of germ cells.

### 2.5 Analysis of DGLA composition in nematodes

Fatty acid composition was analyzed by gas chromatography/mass spectrometry (GC/MS) using the fatty acid methyl ester (FAME) method^[25]^. For each sample, roughly 500 worms were collected in water, allowed to settle, and most of the water removed before freezing the worm pellet. 2.5% sulfuric acid in methanol was added to frozen pellets and then incubated at 70 °C for one hour in a glass tube. FAMEs were extracted in hexane and separated using an Agilent 7890 GC/5975C MS in scanning ion mode equipped with a SP-1380 column. Relative amounts of DGLA/total lipids in nematodes for all DGLA supplementation experiments are reported in Table S1.

## 3. Results

### 3.1 Vitamin B12 rescues DGLA-induced ferroptosis via methionine synthase

As demonstrated previously, *C. elegans* grown on *E. coli* OP50 became sterile when dietarily supplemented with DGLA (Figure 1a)^[22,23]^. Interestingly, when worms were grown on *E. coli* HT115, the bacterial host for RNAi-feeding experiments, the incidence of germ cell death phenotype induced by DGLA was greatly reduced (Figure 1a). While there are likely to be multiple differences between the *E. coli* OP50 and HT115 strains, one well-documented difference is the levels of vitamin B12, with the OP50 strain considered “deficient” in vitamin B12, and B12 measurements showed the HT115 strain contains 2–3-fold higher levels of vitamin B12^[20]^, like mammals, *C. elegans* relies on dietary vitamin B12, and B12 levels in *C. elegans* feeding on HT115 are 3–5-fold higher than in worms feeding on OP50^[20]^. Previous studies demonstrated dietary vitamin B12 impacts *C. elegans* growth, lifespan, reproduction, and stress responses^[11,12,20,21,26]^.

To test the hypothesis that low levels of vitamin B12 lead to susceptibility of germ cells to DGLA-induced ferroptosis in *C. elegans*, worms grown on *E. coli* OP50 were treated with DGLA as previously described^[22,23]^ and exposed to a range of vitamin B12 (cyanocobalamin) doses. There was a striking and highly significant reduction in the levels of DGLA-induced ferroptosis (Figure 1b), indicating vitamin B12 supplementation was sufficient to protect from ferroptosis in wild type worms. Lipid analysis revealed that the addition of vitamin B12 did not reduce the incorporation of DGLA into worm lipids (Table S1). Additionally, DAPI staining revealed worms treated with DGLA alone demonstrated clear loss of germ cells (Figure 1c). However, the germline morphology of wild type worms treated with DGLA and vitamin B12 was largely rescued, with the majority showing a fertile and phenotypically normal germline (Figure 1c). Together, these data demonstrate vitamin B12 supplementation is sufficient to suppress DGLA-induced ferroptosis.

Vitamin B12 is a cofactor for two enzymes. The first, methylmalonase-CoA-mutase (MMCM-1 in *C. elegans*), is a component of the propionate shunt that breaks down propionyl-CoA, a product of branched-chain and odd-chain fatty acid catabolism^[27]^. Additionally, vitamin B12 is required for METR-1 to synthesize methionine from homocysteine as part of the one-carbon cycle (1CC)^[27]^. To test if vitamin B12 rescues ferroptosis through either of these functions, we examined *mmcm-1(ok1637)* and *metr-1(ok521)* mutant strains. When treated with DGLA, *mmcm-1* mutants exhibited sterility due to germ cell death, and the sterility was rescued with vitamin B12 (Figure 1d,e). Conversely, DGLA-induced sterility was not largely rescued by vitamin B12 in *metr-1* mutant worms, and this strain was slightly more sensitive to dietary DGLA compared to wild type (Figure 1d,e).

Because of the reduced levels of vitamin B12 in *E. coli* OP50 compared to *E. coli* HT115^[20]^, we asked if vitamin B12 levels contributed to the observed resistance to DGLA on *E. coli* HT115. To test this, we grew *metr-1* mutant *C. elegans* on NGM plates seeded with either *E. coli* OP50 or *E. coli* HT115 on a medium dose of DGLA. DGLA-treated *metr-1* mutants grown on *E. coli* HT115 were significantly more sensitive to ferroptosis than wild-type worms under the same conditions (Figure 1f,g). Together, these data demonstrate that both supplemented and bacterially derived vitamin B12 protect from DGLA-induced ferroptosis in *C. elegans* through methionine synthase activity.

### 3.2 Phosphatidylcholine synthesis is necessary for vitamin B12-mediated ferroptosis rescue

Methionine synthase function provides essential methyl groups to methyltransferases in the 1CC. Given the necessity of METR-1 to vitamin B12-mediated ferroptosis protection and the increase in sensitivity to ferroptosis in *metr-1* worms, we sought to confirm if downstream 1CC function is also important for vitamin B12-mediated protection from DGLA-induced ferroptosis. We supplemented L-methionine, the product of methionine synthase, with DGLA to wild type and *metr-1 C. elegans* exposed to DGLA. Both worm strains were protected from DGLA-induced sterility, indicating that L-methionine supplementation was sufficient to protect against ferroptosis (Figure 2a,b). To confirm that adequate methyl group availability is required for vitamin B12-mediated protection from ferroptosis, we used *C. elegans sams-1(ok3033)* mutants, deficient in *S*-adenosyl methionine synthase, the primary producer of *S*-adenosylmethionine (SAM) for methyltransferases in *C. elegans*^[28]^. Like *metr-1* mutants, *sams-1* mutants were more sensitive to DGLA compared to wild type worms, and *sams-1* mutants were not significantly rescued by vitamin B12 cotreatment (Figure 2c). The germline morphology of *sams-1* was noticeably different from wild type even without DGLA supplementation (Figure 2d), likely due to the aforementioned reliance of *C. elegans* on SAMS-1 for generation of the methyl group donor, SAM^[28]^. However, vitamin B12 treatment did not make a substantial difference in germline morphology, indicating *sams-1* function is required for vitamin B12 to protect from DGLA-induced ferroptosis (Figure 2d). Together, these findings indicate that methyltransferase activity, enabled by the cofactor vitamin B12, and downstream flux through the 1CC are essential to protect against ferroptosis in *C. elegans*.

In worms, synthesis of PC relies on the phosphobase methylation pathway in which methyltransferases PMT-1 and PMT-2 use the methyl group from SAM to produce the PC precursor molecule phosphocholine from phosphoethanolamine in a mechanism similar to plants^[29]^. Phosphocholine is then converted into PC by using the conventional Kennedy pathway, which enzymatically converts choline into PC through several intermediates^[30,31]^. We found that supplementation of choline, a direct PC precursor required for the phosphomethylation pathway, to wild type and *metr-1 C. elegans* resulted in significant protection of germ cells from DGLA-induced ferroptosis (Figure 2e,f). This indicated that PC synthesis is a key target of the dietary vitamin B12 and methionine rescue of DGLA-induced ferroptosis.

To determine whether efficient PC synthesis was required for ferroptosis protection, we used the reduced function mutant for phosphocholine cytidylyltransferase 1, *pcyt-1(et9)*(C211Y)^[32]^. The function of phosphocholine cytidylyltransferase 1 (PCYT-1) is the conversion of phosphocholine into the direct PC precursor, cytidine diphosphate (CDP)-choline. While *pcyt-1* null mutants are lethal, the *et9*(C211Y) allele confers a partial loss of PC function. We observed that *pcyt-1(et9)* mutants were more sensitive to the effects of DGLA compared to wild type worms and that vitamin B12 cotreatment did not significantly suppress the germline sterility (Figure 2g,h), indicating that sufficient PC synthesis is required for the rescue of DGLA-induced ferroptosis by vitamin B12.

### 3.3 Oleic acid supplementation does not rescue *pcyt-1* sensitivity to dietary DGLA

Based on the observations that monounsaturated fatty acid (MUFA) levels are decreased upon DGLA feeding and that *pcyt-1(et9)* mutants have decreased MUFAs^[32]^, we tested whether supplementation with oleic acid (OA) rescues the sensitivity of *pcyt-1* mutants to dietary DGLA. We found that OA rescues DGLA-supplemented wild type sterility completely, but it only partially rescues the lower dose of DGLA in the *pcyt-1* strain and has little effect on fertility in *pcyt-1* worms treated with higher doses of DGLA (Figure 3a). Interestingly, OA supplementation in the presence of DGLA supplementation led to decreased DGLA content in both wild type and *pcyt-1* worms (Table S1), however, this decrease in DGLA accumulation was not adequate to rescue the sterility phenotypes of *pcyt-1*. Furthermore, the dietary OA did not improve the proportion of fertile *pcyt-1* worms in the absence of DGLA (Figure 3b). This indicates that efficient PC synthesis, particularly PCYT-1 activity, is required for OA rescue of DGLA-induced ferroptosis.

During scoring of the *pcyt-1* DGLA experiments, we noticed distinct germline morphology defects in *pcyt-1* worms without DGLA supplementation, consistent with the fertility defects reported in^[32]^. Most of the worms scored as “fertile” in our assays showed reduced germ cell numbers and sometimes loss of either the anterior or posterior germ cells (Figure 3c). Sterile *pcyt-1* worms often had small clusters of germ cells, similar to the scoring category of sterile (abnormal). Therefore, the germline phenotypes of unsupplemented *pcyt-1* worms and DGLA-fed wild-type worms are similar, suggesting that germ cell death could be occurring in *pcyt-1(et9)* worms.

We previously reported that relative PC levels in life-long DGLA fed worms were not diminished compared to those raised without DGLA^[22]^, and others reported that the PC/phosphatidylethanolamine (PE) ratios were not changed in the *pcyt-1(et9)* mutant strain at 20 °C^[32]^, indicating that the sterility phenotypes of *pcyt-1* mutants or DGLA-treated worms are not due to massive depletion of PC. These fatty acid composition measurements are snapshots of relative lipid levels at one time point. We suspect that efficient PC synthesis is required not only to provide adequate PC levels in membranes, but also to provide substrates for rapid membrane remodeling and membrane repair driven by new PC synthesis. Thus, *pcyt-1(et9)* mutants apparently do not efficiently synthesize enough PC to maintain normal gonadal membranes. This renders them partially sterile and especially sensitive to dietary DGLA in a manner that is unable to be rescued with dietary OA. Future lipidomic studies using dynamic measurements will be necessary to determine the overall rates of PC synthesis, as well as rates of turnover of oxidized lipids in membranes in developing tissues.

## 4. Discussion

The ability of the dietary omega-6 PUFA DGLA to induce germ-cell ferroptosis in *C. elegans* underscores the powerful role of diet in shaping cellular homeostatic networks that maintain membrane integrity and defend against lipid peroxidation, membrane damage, and cell death^[22,23,33]^. Experiments reported here demonstrate that supplementation of vitamin B12, methionine, and choline, protects *C. elegans* germ cells against ferroptotic cell death induced by dietary DGLA. These dietary nutrients contribute to the efficient synthesis of the membrane lipid PC, an essential membrane component assuring membrane stability, membrane protein function, and cellular integrity^[34]^.

Previous work demonstrated the differences in lifespan and reproduction between *C. elegans* fed on *E. coli* OP50 and the vitamin B12-synthesizing bacterium *Comamonas* DA1877^[11,21]^, indicating that dietary vitamin B12 has beneficial effects in *C. elegans*. Neither *C. elegans* nor *E. coli*, the standard laboratory diet of *C. elegans*, contains the biosynthetic enzymes to synthesize vitamin B12. However, *E. coli* encodes transporters to obtain vitamin B12 in the environment, and this vitamin B12 is taken up by *C. elegans* after ingestion of the *E. coli*^[35]^.

Two common *C. elegans* dietary strains, *E. coli* OP50 and HT115, contain different levels of vitamin B12 due to higher expression of the vitamin B12 transporter, TonB, in HT115^[20,36]^, resulting in fertility defects in some *C. elegans* mutants. Notably, the *nhr-114* and *spin-4* mutant strains show sterility when grown on OP50, but not on HT115^[17,35,37,38]^. The sterility has been attributed to a requirement for NHR-114 in the regulation of genes of the 1CC, leading to increased lipid peroxidation and ferroptosis of germ cells^[37]^. The *spin-4* mutants are deficient in the processing of imported vitamin B12 in the lysosome, limiting vitamin B12 in the cytosol for use in PC synthesis^[38]^. Our data support these previously reported fertility outcomes, demonstrating a role for vitamin B12-dependent phosphatidylcholine synthesis in dietary DGLA-induced germ cell death. (Figure 4).

Methyl groups produced in the 1CC are necessary for many processes including DNA methylation, amino acid synthesis, chromatin methylation, and lipid synthesis. SAMS-mediated hypermethylation of promoters of genes encoding ferroptosis regulators such as glutathione peroxidase 4 (GPX4) can alter ferroptosis sensitivity in mammals^[39]^. Furthermore, other epigenetic modifications of RNA, histones, or other proteins are known to influence ferroptosis^[40]^. Recently, dietary methionine restriction rendered glioma cell lines sensitive to RSL3-induced ferroptosis^[41]^, indicating the importance of sufficient dietary sources of methyl groups in ferroptosis protection in mammals.

Our work supports previous studies showing that PC synthesis requires constant synthesis of methyl groups in reproductive *C. elegans*^[28]^. *E. coli* does not produce PC^[42]^, requiring dietary choline and methyl groups to maintain PC levels in membranes. Dietary vitamin B12, choline, and methionine were highly effective in rescuing DGLA-induced ferroptosis, indicating that various ways of restoring methyl groups lead to ferroptosis resistance, likely due to increased PC turnover under ferroptosis-inducing conditions. In previous labeling studies, oxidative stress has been shown to lower the levels of PC in *C. elegans*^[43]^. However, previous research showed that relative PC levels increase in wild type worms after DGLA feeding, and that *pcyt-1* mutants do not exhibit a reduced PC/PE ratio compared to wild type at 20 °C^[22,32]^. Therefore, PC levels appear to be maintained in the worm outside of efficient PC synthesis. We propose that under ferroptosis-inducing conditions, increased methionine and choline metabolites, along with efficient PCYT-1 activity enables higher turnover of PC, leading to the removal of oxidized phospholipids and membrane protection.

A limitation of this study was the lack of dynamic measurements of lipid species due to technical challenges in a small organism, especially the identification of oxidized phospholipid species. Furthermore, identification of epigenetic changes due to methyl group availability was not carried out in this study.

A pivotal finding in unraveling ferroptosis mechanisms was that oxidized arachidonic acid and adrenic acid in PE are associated with ferroptosis^[44]^. Our study demonstrates that overall membrane lipid homeostasis, especially efficient PC synthesis, is crucial for cells to maintain membrane integrity and ferroptosis protection. Other recent studies highlight the importance of PC in ferroptosis regulation in *C. elegans*^[38]^ as well as roles for PC-PUFAs and oxidized PC species in mammalian cells^[45,46]^. Even though PC contains highly unsaturated long chain PUFAs that contribute to ferroptosis susceptibility, the Lands Cycle, including lipid remodeling enzymes such as lysophosphatidylcholine acyltransferase 3 (LPCAT3), ensures constant turnover and removal of peroxidized lipids^[47]^. With the understanding that PC homeostasis is critical for protection against ferroptosis, targeting methionine, choline, and lipid synthesis in tumor microenvironments has the potential to sensitize cancer cells to therapeutics that induce ferroptosis.

## 5. Conclusions

Sufficient dietary vitamin B12, methionine, and choline are required for PC synthesis to protect germ cells from ferroptosis triggers such as dietary DGLA. Membrane maintenance is crucial to promote protection against stressors that induce ferroptosis.

## Supplementary Material

Supplementary materials

The supplementary material for this article is available at: Supplementary materials.

## Figures and Tables

**Figure 1.**
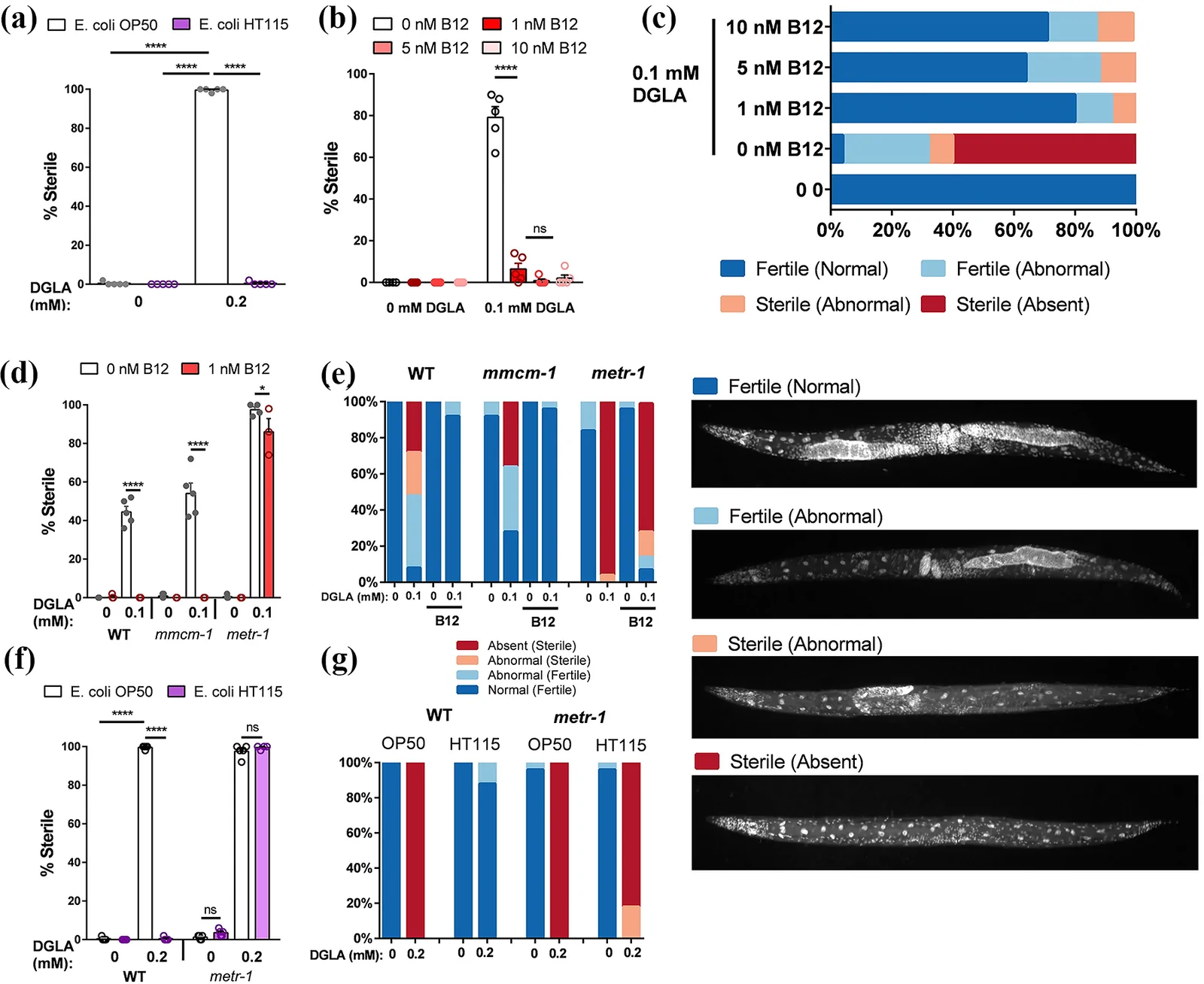
Vitamin B12 rescues DGLA-induced Ferroptosis. (a) Sterility percentage of five technical replicates of 50-worm populations after treatment with DGLA and grown on *E. coli* OP50 or *E. coli* HT115. Statistical analysis was performed using a one-way ANOVA with Tukey’s multiple comparisons test; (b) Sterility percentages after treatment with 0.1 mM DGLA and a range of vitamin B12 doses; (c) Germline morphology analysis of wildtype worms treated with 0.1 mM DGLA and various doses of vitamin B12 (*n* = 25 worms/treatment); (d) Sterility percentage of wild type, *mmcm-1*, and *metr-1* worm populations after treatment with 0.1 mM DGLA and 1 nM vitamin B12; (e) Germline morphology analysis of worms shown in panel d (*n* = at least 14, typically 25 worms/treatment); (f) Sterility percentage of wild type and *metr-1* mutant worms supplemented with 0.2 mM DGLA grown on *E. coli* OP50 or HT115; (g) Germline morphology analysis of worms shown in panel f (*n* = at least 22, typically 25 worms/treatment). Statistical analysis was performed using GraphPad Prism using a two-way ANOVA with Tukey’s multiple comparisons test. DGLA: dihomo-gamma-linolenic acid; WT: wild type; ANOVA: analysis of variance.

**Figure 2.**
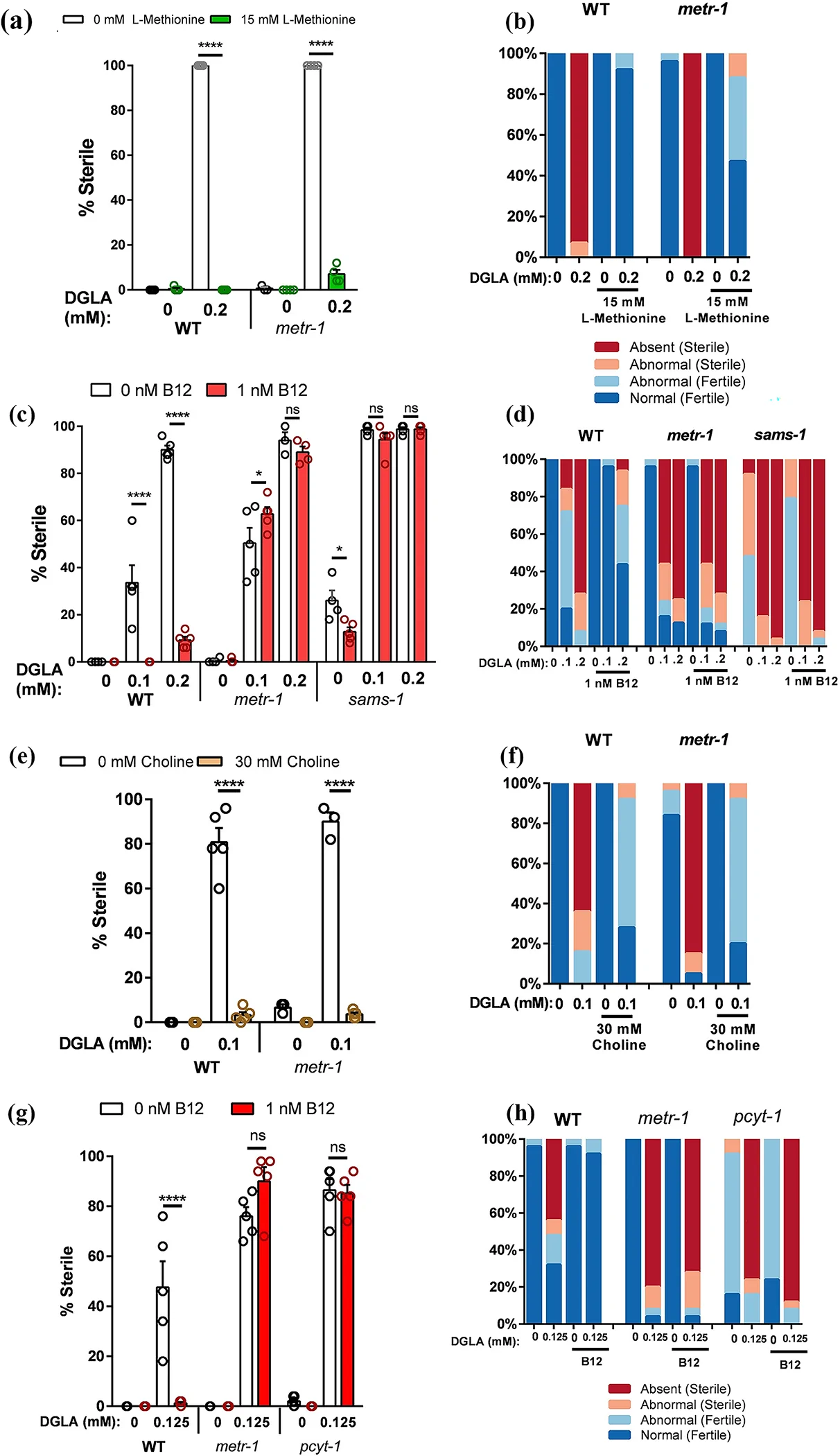
The One-Carbon Cycle is essential for vitamin B12-mediated ferroptotic suppression. (a) Sterility percentage of wild type or *metr-1* exposed to DGLA and L-methionine; (b) Germline morphology analysis of worms shown in panel a (*n* = at least 14, typically 25 worms/treatment); (c) Sterility percentage of wild type, *metr-1,* and *sams-1* exposed to DGLA and vitamin B12; (d) Germline morphology analysis of worms shown in panel c (*n* = at least 14, typically 25 worms/treatment); (e) Sterility percentage of wild type or *metr-1* mutant worm population exposed to DGLA and choline chloride; (f) Germline morphology analysis of worms shown in panel e (*n* = at least 20, typically 25 worms/treatment); (g) Sterility percentage of wild type, *metr-1*, or *pcyt-1* exposed to DGLA and vitamin B12; (h) Germline morphology analysis of worms shown in panel g (*n* = 25 worms/treatment). Statistical analysis was performed using GraphPad Prism via 2-way ANOVA with Tukey’s multiple comparisons test. DGLA: dihomo-gamma-linolenic acid; WT: wild type.

**Figure 3.**
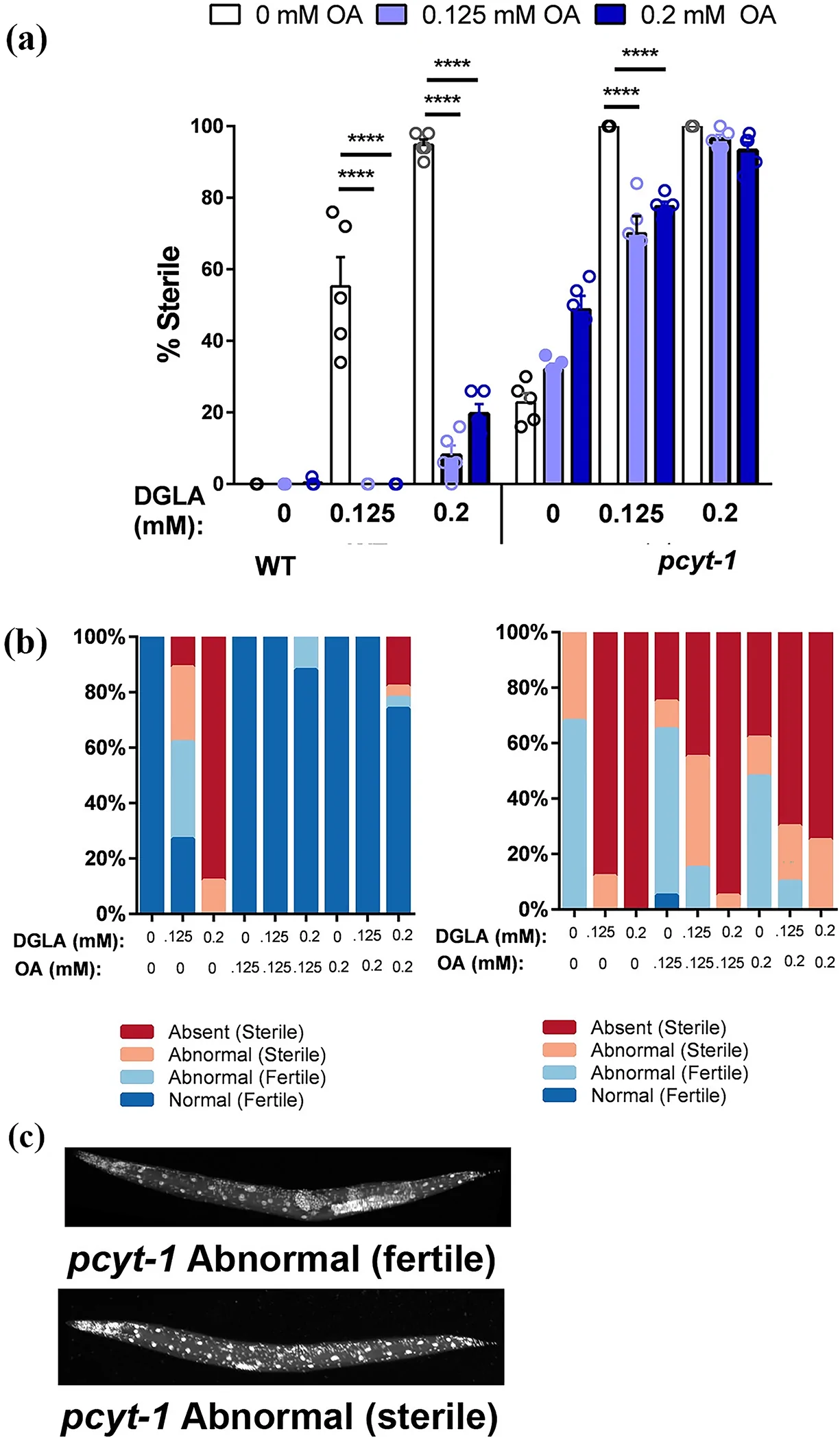
Dietary oleate does not rescue germline defects in *pcyt-1* mutants. (a) Sterility percentage of five technical replicates of 50-worm populations after treatment with various doses of DGLA and OA. Statistical analysis was performed using a one-way ANOVA with Tukey’s multiple comparisons test; (b) Germline morphology analysis of worms shown in panel a (*n* = at least 20, typically 25 worms/treatment); (c) Representative DAPI images of sterile *pcyt-1* worms showing germline defects in the absence of DGLA treatment. DGLA: dihomo-gamma-linolenic acid; OA: oleic acid; DAPI: 4′,6-diamidino-2-phenylindole; ANOVA: analysis of variance.

**Figure 4.**
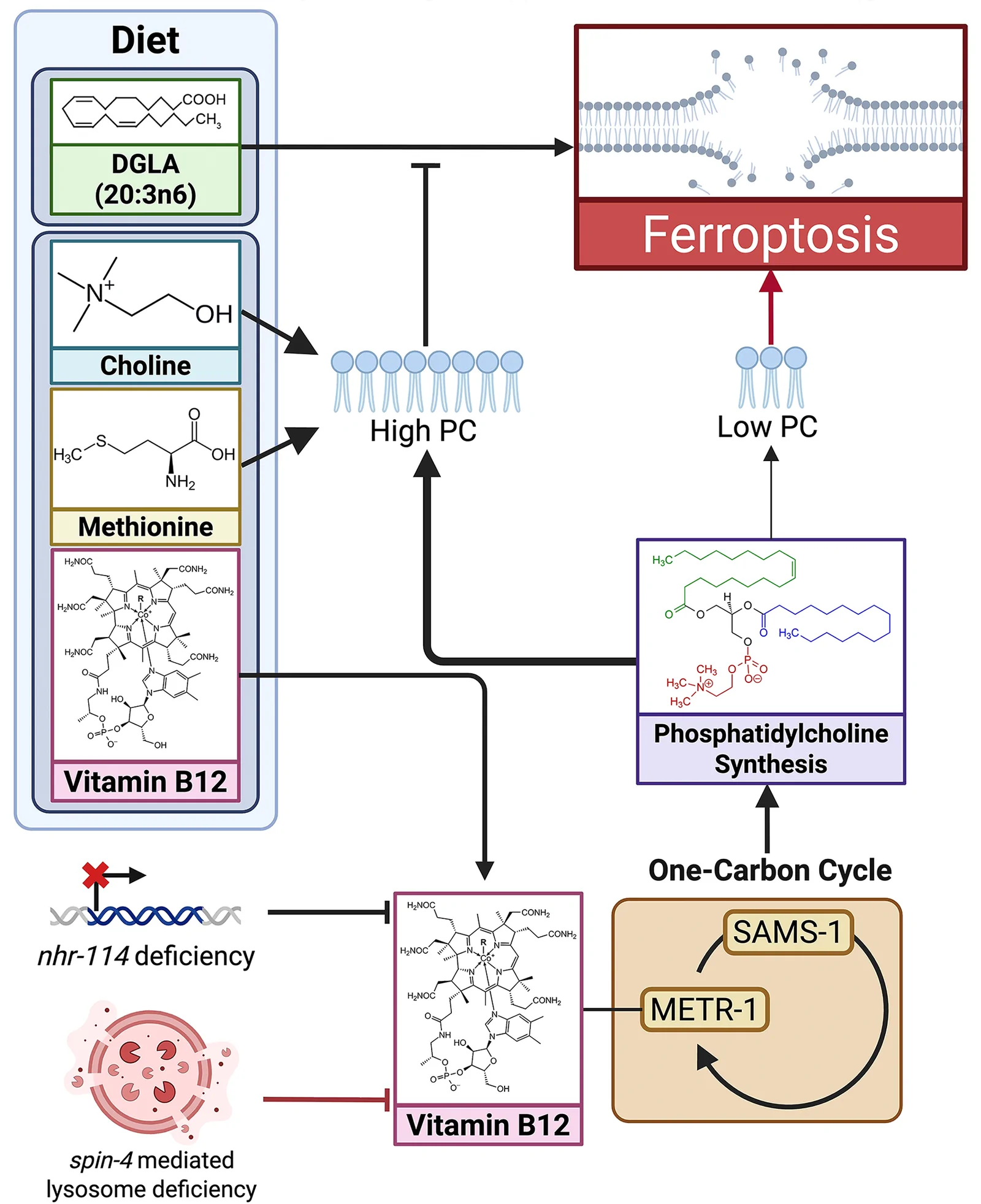
A model depicting important dietary nutrients that influence germ cell ferroptosis in *C. elegans*. Dietary DGLA causes sterility due to germ cell ferroptosis when worms grow on a low B12 diet. The sterility is rescued by dietary vitamin B12, methionine, or choline. Mutant strains lacking *nhr-114* and *spin-4* mutant worms have defects in vitamin B12 assimilation and undergo germ cell ferroptosis on low B12 diets without DGLA supplementation. Created in BioRender. Mortensen, M. (2026) https://BioRender.com/q4q7qcm. DGLA: dihomo-gamma-linolenic acid; *C. elegans*: *Caenorhabditis elegans.* METR-1: methionine synthase; PC: phosphatidylcholine.

## Data Availability

The data that support the findings of this study are available from the corresponding author upon reasonable request.
