# Supplementary material for: Dietary DGLA-induced ferroptosis is suppressed by vitamin B12-mediated phosphatidylcholine synthesis
Source: Ferroptosis Oxid Stress. Author manuscript; Available in PMC 2026 Nov 11. (PMC13750769; doi:10.70401/fos.2026.0045)
Supplement: Supplementary file 1 [file NIHMS2216425-supplement-Supplementary_materials.zip › Table S1.pdf]

Ferroptosis and Oxidative Stress
https://doi.org/10.70401/fos.2026.0045

Supplementary information

Dietary DGLA-induced ferroptosis is suppressed by vitamin B12-mediated phosphatidylcholine synthesis

Madison S. Mortensen, Paige E. Silvia, Carissa Perez Olsen, Jennifer L. Watts

Table S1: Relative concentration of DGLA (% of total lipid) in nematodes measured with GC/MS

% DGLA/total fatty acids relating to Figure 1A and Figure 1E				
**Genotype**	**Bacteria**	**0 mM DGLA**	**0.2 mM DGLA**	
N2	OP50	4.46%	17.32%	
	HT115	5.21%	10.61%	
metr-1(ok521)	OP50	3.98%	12.65%	
	HT115	3.52%	10.81%	
**%DGLA/total fatty acids relating to Figure 1B**				
**Genotype**	**Cotreatment**	**0 mM DGLA**	**0.1 mM DGLA**	
N2	0 nm B12	3.51%	7.07%	
	1 nm B12	4.61%	9.90%	
	5 nm B12	4.62%	10.14%	
	10 nm B12	5.51%	10.19%	
**%DGLA/total fatty acids relating to Figure 1D**				
**Genotype**	**Cotreatment**	**0 mM DGLA**	**0.1 mM DGLA**	
N2	0 nm B12	3.70%	7.45%	
	1 nm B12	5.52%	11.38%	
metr-1(ok521)	0 nm B12	4.25%	7.17%	
	1 nm B12	3.69%	6.50%	
mmcm-1(ok1637)	0 nm B12	4.00%	8.03%	
	1 nm B12	4.42%	10.59%	
**DGLA %/total lipids relating to Figure 2A**				
**Genotype**	**Cotreatment**	**0 mM DGLA**	**0.1 mM DGLA**	
N2	0 mm L-Methionine	4.41%	9.52%	
	15 mm L-Methionine	3.98%	7.74%	
metr-1(ok521)	0 mm L-Methionine	2.71%	13.28%	
	15 mm L-Methionine	3.73%	6.14%	

**DGLA %/total lipids relating to Figure 2B**				
**Genotype**	**Cotreatment**	**0 mM DGLA**	**0.1 mM DGLA**	**0.2 mM DGLA**
N2	0 nm B12	3.99%	6.87%	11.38%
	1 nm B12	4.92%	10.26%	8.62%
metr-1(ok521)	0 nm B12	3.62%	6.00%	8.47%
	1 nm B12	3.55%	6.06%	8.73%
sams-1(ok3033)	0 nm B12	4.27%	7.13%	8.41%
	1 nm B12	5.52%	6.72%	
**%DGLA/total fatty acids relating to Figure 2C**				
**Genotype**	**Cotreatment**	**0 mM DGLA**	**0.2 mM DGLA**	
N2	0 mm Choline	4.37%	11.82%	
	30 mm Choline	6.31%	13.03%	
metr-1 (ok521)	0 mm Choline	3.32%	10.33%	
	30 mm Choline	5.54%	20.12%	
**%DGLA/total fatty acids relating to Figure 2D**				
**Genotype**	**Cotreatment**	**0 mM DGLA**	**0.125 mM DGLA**	
N2	0 nm B12	3.41%	6.35%	
	1 nm B12	4.13%	9.87%	
metr-1(ok521)	0 nm B12	2.97%	6.28%	
	1 nm B12	3.60%	5.55%	
pcyt-1(et9)	0 nm B12	3.87%	6.52%	
	1 nm B12	4.08%	5.37%	
**%DGLA/total fatty acids relating to Figure 3A**				
**Genotype**	**Cotreatment**	**0 mM DGLA**	**0.125 mM DGLA**	**0.2 mM DGLA**
N2	0 nm OA	3.5%	7.7%	10.9%
N2	0.125 nm OA	3.7%	4.2%	5.6%
N2	0.2 nm OA	3.2%	4.2%	5.1%
pcyt-1(et9)	0 nm OA	3.6%	6.6%	7.6%

pcyt-1(et9)	0.125 nm OA	3.6%	4.7%	5.0%
pcyt-1(et9)	0.2 nm OA	3.4%	4.0%	5.3%
